# Reply: Evaluating the activity of temsirolimus in neuroendocrine cancer

**DOI:** 10.1038/sj.bjc.6603514

**Published:** 2006-12-12

**Authors:** I Duran, M J Moore, L L Siu

**Affiliations:** 1 Princess Margaret Hospital Phase II Consortium, Toronto, Canada M5G 2M9

**Sir**,

We appreciate the interest of O'Donnell *et al* in our report on temsirolimus in neuroendocrine cancers (NEC). They suggest that based on the tumour control rate observed in our study, temsirolimus may have activity in advanced NEC ‘beyond the natural course of disease’. We completely agree with this observation. In fact, as discussed in our manuscript, at the end of stage I in this two-staged trial, we had amended the protocol despite not meeting the originally set criteria, and proceeded to stage II based on the observed decrease in tumour progression rate and clinical benefit in some patients. We concluded the manuscript by declaring that temsirolimus appears to have modest activity in NEC, and advocating the development of combination studies with this agent.

We also share the enthusiasm of O'Donnell *et al* for efficient clinical trial designs to evaluate the activity of new molecularly targeted compounds. However, we would respectfully disagree with their statement that this is best achieved by abandoning single-arm phase II studies.

Phase II trials play a pivotal screening role in the drug development process (Mariani and Marubini, 2000; Taylor *et al*, 2006). Given that patient and financial resources are precious, and there is a pressing need to rapidly bring true advances to the clinic, efficient phase II trial designs that reliably retain promising agents while quickly discard inactive ones are critical. This is particular true when we have such a wide range of novel agents undergoing evaluation. Traditional single-arm phase II trials that focused solely on response might underestimate the cytostatic activity of some molecular targeted agents; however including tumour control within the primary end point can overcome this limitation, as can referencing the tumour control rates to other studies of less-active agents in the same population (as was done by O'Donnell *et al* in coming to the conclusion that temsirolimus does have some activity against NEC based upon our study results). In our study, after the accrual of 37 patients over 18 months, we were able to draw useful conclusions about the antitumour activity of temsirolimus in NEC, thus reaffirming that single-arm phase II trials remain a good choice for promptly determining if a drug should advance to further development.

The randomised discontinuation design has been proposed as an alternate more complex option to evaluate the efficacy of cytostatic drugs (Ratain *et al*, 2006). This design represents an appealing alternative with some limitations (Freidlin and Simon, 2005). These studies tend to be much larger than other phase II designs; as an example, 368 patients were entered on a clinical trial of carboxyaminoimidazole in renal cell carcinoma that used the randomised discontinuation design and concluded that the drug was inactive (Stadler *et al*, 2005). Given the multitude of interesting options for renal cell cancer under evaluation, a design that requires over 350 patients to conclude that a drug is inactive lacks the efficiency we are all seeking. As an interesting comparison, two multikinase inhibitors, sorafenib and sunitinib, have now been approved for advanced renal cell cancers. The phase II development of sorafenib utilised the randomised discontinuation design and enrolled 202 patients, 36% of patients had tumour shrinkage of ⩾25% and 32% achieved stable disease at 12 weeks (Ratain *et al*, 2006). The phase II development of sunitinib utilised a single-arm phase II design and enrolled 106 patients, 34% of patients had partial responses by RECIST criteria and 29% achieved stable disease for ⩾3 months (Motzer *et al*, 2006). Both trials led to further phase III evaluations due to the promising activity detected by their respective phase II designs.

Lastly, O'Donnell *et al* draw analogies between renal cell carcinoma and NEC. One needs to be cautious in extrapolating from an experience in a tumour that has a different biology, natural history and response to therapy.
